# Supplementary material for: The Systems Biology Research Tool: evolvable open-source software
Source: BMC Syst Biol. 2008 Jun 29;2:55. doi: 10.1186/1752-0509-2-55 (PMC2446383; doi:10.1186/1752-0509-2-55)
Supplement: Additional file 1 — SBRT Archive. An archive of the current version of the Systems Biology Research Tool. [file 1752-0509-2-55-S1.zip › sbrt-1.4.0/doc/developers_guide/api/sbrt/kernel/fba/class-use/ReversibleRxn.html]

Uses of Class sbrt.kernel.fba.ReversibleRxn


|  |  |  |
| --- | --- | --- |
| **Overview** \| **Package** \| **Class** \| **Use** \| **Tree** \| **Deprecated** \| **Index** \| **Help** | |  |
| PREV   NEXT | **FRAMES**    **NO FRAMES**     **All Classes** |  |


---


## **Uses of Class sbrt.kernel.fba.ReversibleRxn**

| Packages that use ReversibleRxn | |
| --- | --- |
| **sbrt.shell.text.chem** | Provides classes and interfaces for defining formats for chemistry related objects. |

Uses of ReversibleRxn in sbrt.shell.text.chem

| Methods in sbrt.shell.text.chem that return ReversibleRxn | |
| --- | --- |
| `ReversibleRxn` | `RevRxnFormatV1.parse(java.lang.String reaction)`             Parses the provided string and returns its corresponding chemical reaction. |
| `ReversibleRxn` | `RevRxnFormatV2.parse(java.lang.String reaction)`             Parses the provided string and returns its corresponding chemical reaction. |

| Methods in sbrt.shell.text.chem with parameters of type ReversibleRxn | |
| --- | --- |
| `java.lang.String` | `RevRxnFormatV1.format(ReversibleRxn rxn)`             Returns a formatted string representation of the provided reversible chemical reaction. |
| `java.lang.String` | `RevRxnFormatV2.format(ReversibleRxn rxn)`             Returns a formatted string representation of the provided reversible chemical reaction. |

---


|  |  |  |
| --- | --- | --- |
| **Overview** \| **Package** \| **Class** \| **Use** \| **Tree** \| **Deprecated** \| **Index** \| **Help** | |  |
| PREV   NEXT | **FRAMES**    **NO FRAMES**     **All Classes** |  |


---
